# Impacts of Geomagnetic and Man-Made Electromagnetic Fields on Heart Rate and Its Variability in Healthy Adults: A Scoping Review

**DOI:** 10.7759/cureus.114429

**Published:** 2026-08-12

**Authors:** Taha Fadlou Allah, Brian Wexler, Daniel M Green, Yash N Patel, Jason Chen, Harvey N Mayrovitz

**Affiliations:** 1 Medicine, Nova Southeastern University Dr. Kiran C. Patel College of Osteopathic Medicine, Davie, USA; 2 Medical Education, Nova Southeastern University Dr. Kiran C. Patel College of Allopathic Medicine, Davie, USA

**Keywords:** autonomic nervous system, bioelectromagnetic, cardiovascular physiology, electromagnetic fields, geomagnetic activity, geomagnetic field, heart rate, heart rate variability, magnetic fields

## Abstract

Geomagnetic and man-made electromagnetic fields (EMFs) have been reported to influence blood pressure and to represent a cardiovascular risk factor for various conditions. However, the effects of these fields on heart rate (HR) and heart rate variability (HRV) remain inconclusive, with reports both supporting and refuting these effects. This scoping review aims to synthesize current evidence on the impact of exposure to geomagnetic and man-made EMFs on HR and HRV in healthy adults. A systematic search of Ovid MEDLINE, Embase, and Web of Science was conducted using the Population-Concept-Context (PCC) framework, following the Joanna Briggs Institute (JBI) methodology for scoping reviews and the Preferred Reporting Items for Systematic Reviews and Meta-Analyses extension for Scoping Reviews (PRISMA-ScR) guidelines. Eligible studies included English-language, peer-reviewed observational, experimental, or analytical cross-sectional designs published from January 2000 to June 2026 that assessed HR or HRV responses to these fields in healthy or asymptomatic adults. Database searching was supplemented by reference review and an updated search of the same databases. After two-stage screening and duplicate removal, 29 studies were included, which varied substantially in exposure type, field intensity, exposure duration, HRV metrics, sample size, and analytic approach. Reported effects were inconsistent and were often limited to transient changes in selected HRV indices rather than consistent changes in HR. Geomagnetic and local magnetic field studies more often reported associations with HRV, hemodynamic variation, or HR/HRV synchronization, but these findings were heterogeneous and of uncertain clinical significance. Overall, evidence from the studies reviewed does not confirm a consistent effect of geomagnetic or man-made EMF exposure on either HR or HRV in otherwise healthy adults.

## Introduction and background

Exposure to geomagnetic fields is inevitable, and exposure to man-made electromagnetic fields (EMFs) is extremely prevalent in our society and an unavoidable aspect of modern life. Understanding how these fields influence human physiology is increasingly important, as they may affect cardiovascular parameters such as heart rate (HR) and heart rate variability (HRV), which are key indicators of autonomic nervous system (ANS) balance and cardiovascular health. Despite increased public concern and ongoing scientific inquiry, findings on these effects remain inconsistent across the literature.

Geomagnetic fields are naturally generated by the Earth’s core and modulated by solar activity and space weather. Fluctuations in these fields have been linked to changes in various aspects of cardiovascular physiology [1-4]. Recent reviews have also discussed possible associations between geomagnetic activity and blood pressure variation [5] and broader cardiovascular outcomes [6], although the available evidence remains heterogeneous and, in some cases, inconclusive. Several studies have reported associations between geomagnetic disturbances and alterations in HR and HRV, suggesting a possible modulation of autonomic activity [1]. For instance, increases in geomagnetic activity have been correlated with elevated HRV and parasympathetic tone, while geomagnetic storms have been associated with transient cardiovascular changes [7]. Although these observations suggest a physiological connection, the strength and clinical relevance of such associations remain uncertain.

Artificial magnetic fields, including those produced by medical devices such as magnetic resonance imaging (MRI) scanners and occupational equipment, have been studied for their potential cardiovascular effects [8]. Some studies have suggested that static magnetic fields may increase blood flow and reduce blood viscosity, whereas controlled low-frequency (LF) magnetic field exposure has produced little or no change in mean arterial pressure [9,10]. Additional research has analyzed alterations in HRV parameters following magnetic field exposure, suggesting potential modulation of cardiac autonomic control [10-13]. Conversely, other studies have found no significant changes in HR or HRV, highlighting variability in study designs, exposure intensities, and measurement methods [13,14].

The widespread use of wireless devices has increased interest in the health implications of EMF exposure, a topic that has been extensively studied [15,16]. While organizations such as the Environmental Working Group and the World Health Organization have acknowledged potential physiological effects, the evidence for adverse cardiovascular outcomes remains limited [15,16]. Experimental studies examining extremely low-frequency (ELF) or radiofrequency (RF) EMFs have produced conflicting results, with some reporting subtle changes in HRV and others finding no measurable impact on the ANS [12]. Prolonged exposure to EMFs has been associated with an increased risk of arrhythmia-related mortality in some cohorts, although causality remains unproven [17]. Overall, current evidence suggests that these fields may affect HR and HRV under certain conditions, but the findings remain inconsistent [18].

Given the unavoidable exposure to geomagnetic fields and the exponential increase in exposure to various EMFs, it is important to understand the potential impact of these fields on multiple aspects of human physiology. Several studies have reported that changes in the geomagnetic field or cosmic ray intensity affect HR parameters in hospitalized patients or in others with various cardiovascular conditions [19-21]. However, the important question of whether such effects occur in otherwise healthy adults remains open. Thus, the present study focuses on the potential effects of geomagnetic disturbances or man-made EMFs on HR and HRV in healthy adults. Specifically, we ask what we can conclude from the overall relevant literature regarding this issue. For this purpose, the available evidence is synthesized to summarize and map the extent, nature, and quality of the research.

## Review

Methods

Study Design and Search Strategy

A comprehensive search was performed in September 2024 and updated in June 2026 across Embase, Ovid MEDLINE, and Web of Science, following the Preferred Reporting Items for Systematic Reviews and Meta-Analyses extension for Scoping Reviews (PRISMA-ScR) guidelines [22]. The protocol adhered to the Joanna Briggs Institute (JBI) methodology for scoping reviews [23]. The Population-Concept-Context (PCC) framework guided the search strategy, with the population defined as healthy or asymptomatic adults, the concept as the effects of geomagnetic or man-made EMF exposures on HR or HRV, and the context as global studies involving human participants. A preliminary search of MEDLINE, the Cochrane Database of Systematic Reviews, PROSPERO, and JBI Evidence Synthesis identified no ongoing or completed scoping or systematic reviews on this topic. The search strategy was adapted for each database using controlled vocabulary (e.g., MeSH and Emtree terms) combined with Boolean operators (AND/OR) to capture relevant synonyms and related terms. The full database-specific search strategies used in the September 2024 search are provided in Table 1.

**Table 1:** Search strategies used across Embase, Ovid MEDLINE, and Web of Science Core Collection.

Search #	Search terms used	Number of articles
Embase		
#1	'heart rate':ab,ti,kw OR 'heart rate variability':ab,ti,kw	261,332
#2	'magnetic':ab,ti,kw OR 'geomagnetic':ab,ti,kw OR 'electromagnetic':ab,ti,kw	809,653
#3	'normal human'/exp OR 'asymptomatic':ab,ti,kw	809,406
#4	#1 AND #2 AND #3	511
Ovid MEDLINE		
#1	('heart rate' OR 'heart rate variability').mp.	185,561
#2	('magnetic' OR 'geomagnetic' OR 'electromagnetic').ab,ti,kf.	688,445
#3	('healthy adult' OR 'healthy individual' OR 'asymptomatic' OR 'healthy participant*' OR 'healthy volunteer*' OR 'healthy people' OR 'healthy subject*').mp.	446,184
#4	#1 AND #2 AND #3	311
Web of Science Core Collection		
#1	TI=('heart rate*' OR 'heart rate variability*')	47,025
#2	TS=('magnetic' OR 'geomagnetic' OR 'electromagnetic')	2,130,333
#3	TS=('healthy adult' OR 'healthy individual' OR 'asymptomatic' OR 'healthy participant*' OR 'healthy volunteer*' OR 'healthy people' OR 'healthy subject*')	987,142
#4	#1 AND #2 AND #3	105

To account for literature published after the initial search, an updated database search was conducted in June 2026 using the same search strategy in Embase, Ovid MEDLINE, and Web of Science. This updated search screened records published after the September 2024 search date through June 2026. In addition, supplemental expert-identified and reference-list searching was performed in June 2026 to identify potentially relevant studies not captured by the original database search. Records identified through these supplemental methods were screened using the same inclusion and exclusion criteria as those used in the original search.

Eligibility Criteria

Studies were included if they were peer-reviewed, experimental, observational, or cross-sectional in design and the results were published in English from the year 2000 through June of 2026. To be included, the studies must have investigated the effects of geomagnetic fields, magnetic fields, or EMFs on HR or HRV in healthy or asymptomatic adult populations. Studies were excluded if they were reviews, editorials, letters, conference abstracts without full reports, case reports or case series, animal or in vitro studies, pediatric studies, studies involving participants with known pre-existing disease without separable healthy-adult data, studies without HR/HRV outcomes, or articles unavailable in full text.

Screening and Selection of Sources of Evidence

The initial database search yielded 927 records, of which 164 duplicates were removed, leaving 763 unique citations for screening. Five reviewers independently screened all titles and abstracts for relevance. Most records excluded at title/abstract screening did not evaluate eligible field exposures in humans, did not report HR or HRV outcomes, or did not meet the healthy adult inclusion criteria. Seventy-five studies were retained for full-text review by two independent reviewers, with a third reviewer serving as a tiebreaker when disagreements arose. Consensus was achieved through discussion. A total of 54 studies were excluded at the full-text stage for the following reasons: ineligible study design, absence of relevant field exposure, inaccessible full texts, lack of HR or HRV measurement, duplicate reporting, or failure to meet the healthy/asymptomatic adult population criterion. The initial search and screening process resulted in 21 included studies.

**Figure 1:**
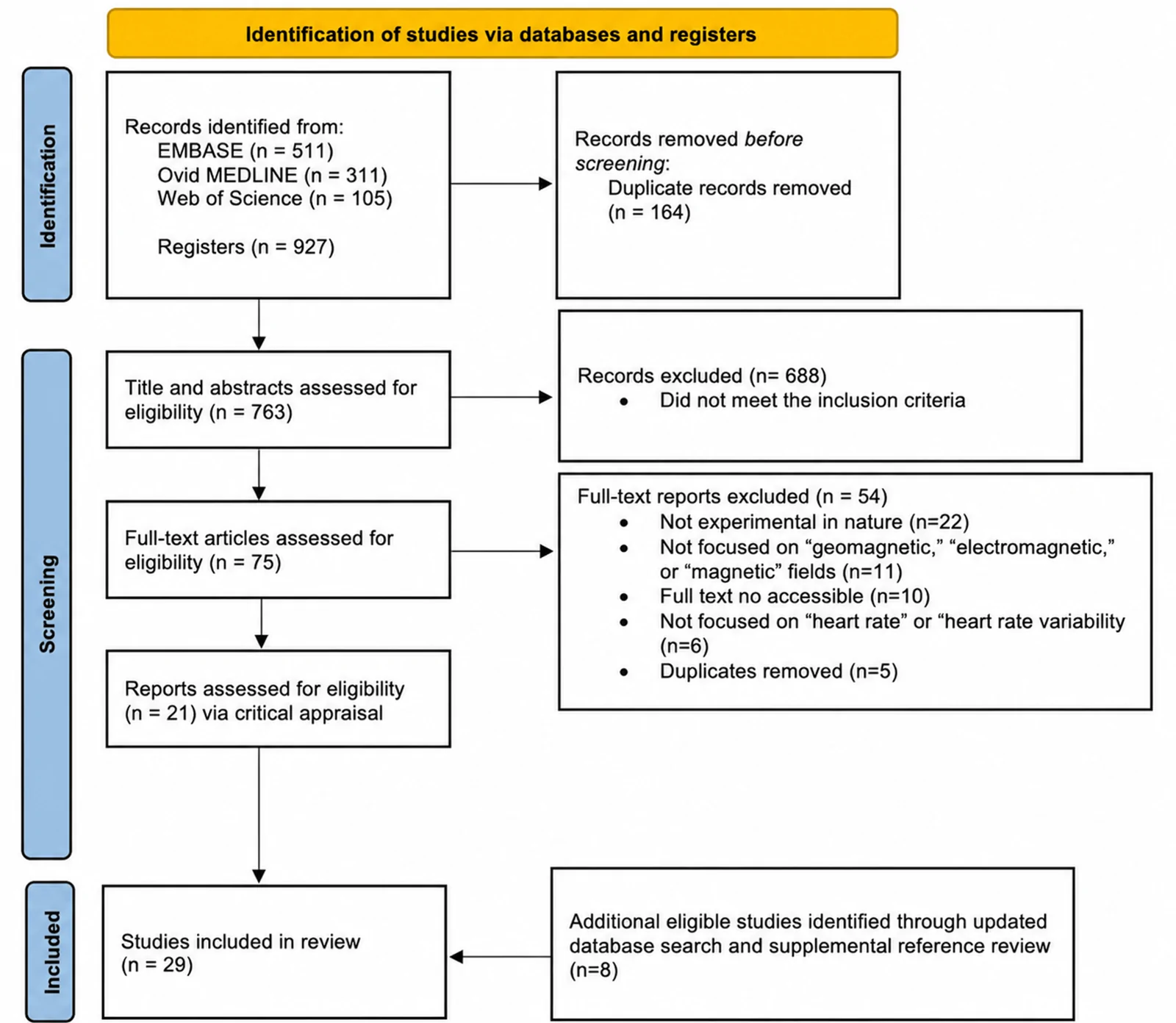
PRISMA flow diagram of study identification and selection. PRISMA = Preferred Reporting Items for Systematic Reviews and Meta-Analyses.

The updated database search performed on June 20, 2026, yielded 76 records for screening, of which one study met the inclusion criteria. Supplemental expert-identified and reference-list searching identified seven additional eligible studies. Together, these processes resulted in a final total of 29 included studies. The study-selection process, including records identified through database searching and supplemental expert/reference-list review, is summarized in the PRISMA flow diagram shown in Figure 1.

Data Charting and Synthesis of Results

A standardized data-charting form was developed and pilot-tested by two reviewers to ensure consistency. Extracted variables included study design, population characteristics, exposure type and parameters, measured HR or HRV indices, and key outcomes. Both reviewers independently verified and extracted all data prior to synthesis. Results were summarized descriptively and grouped into four exposure categories: RF/mobile phone exposure, ELF or pulsed EMF, static magnetic field exposure, and geomagnetic field exposure. Findings were organized in a tabular format to facilitate comparison across all studies.

Critical Appraisal

Methodological quality and risk of bias were assessed using the JBI Critical Appraisal Tools appropriate to each study design [23]. Each included study was independently reviewed by five authors, and disagreements were resolved through discussion until a consensus was reached. Criterion-level judgments were recorded as “Yes”, “No”, “Unclear”, or “Not applicable”.

Because the JBI tools are design-specific and do not specify a universal numerical threshold for an overall rating, the review team applied a prespecified synthesis rule to translate these criterion-level judgments into an overall level of methodological concern for each study. Low concern was assigned when no critical methodological limitation was identified, and any remaining uncertainty was confined to noncritical items. Moderate concern was assigned when one or more limitations or unclear judgments could plausibly affect interpretation, but no single flaw, or combination of flaws, was judged likely to invalidate the study's principal findings. High concern was assigned when at least one critical, design-defining limitation substantially threatened validity, or when a combination of serious limitations was judged likely to materially bias interpretation. These ratings informed the interpretation of findings but were not used to exclude studies, consistent with standard scoping review practice.

Of the 29 included studies, four were judged to have low methodological concern, 11 moderate concern, and 14 high concern. Notably, an overall high-concern rating was assigned if a study exhibited at least one critical, design-defining methodological flaw. High methodological concern was most frequent among geomagnetic studies, affecting eight of the 14 studies in that category. Across all included literature, the most common reasons for high concern ratings were inadequate control conditions, incomplete control of environmental or behavioral confounding, and the use of small or non-independent participant samples. The distribution of appraisal ratings by exposure category is summarized in Table 2. Study-specific criterion-level judgments are presented in Supplementary Tables included in the Appendices.

**Table 2:** Summary of methodological appraisal by exposure category. RF = radiofrequency; ELF = extremely low frequency.

Exposure category	Low concern	Moderate concern	High concern	Total
RF/mobile phone	0	3	3	6
ELF/pulsed electromagnetic fields	2	3	2	7
Static magnetic fields	1	0	1	2
Geomagnetic fields	1	5	8	14
Total	4	11	14	29

Results

A total of 29 studies were included in this scoping review, encompassing investigations of exposure to geomagnetic and man-made EMFs and their effects on HR and HRV in healthy or asymptomatic adults. Study designs, exposure parameters, and outcome measures varied. Included studies covered acute RF exposures (e.g., mobile phones or high-frequency EMF), ELF/power-frequency magnetic fields (including occupational and sleep settings), static magnetic fields in the Tesla range, and geomagnetic activity assessed either observationally over days or under laboratory-simulated storm conditions. Study-level exposure conditions, parameters evaluated, and outcomes are summarized in Table 3.

**Table 3:** Summary of included articles. Column 1 = first author, year of publication, reference number. Column 2 = exposure type and magnitude used in the study. Column 3 = sample size. Column 4 = parameters evaluated in the study. Column 5 = main outcome of the study. The following abbreviations and units are used in this table: min = minute; GSM = Global System for Mobile Communications; MHz = megahertz; HRV = heart rate variability; HR = heart rate; SDNN = standard deviation of normal-to-normal intervals; VLF = very-low-frequency; HF = high frequency; LF/HF = ratio of low-frequency to high-frequency power; LF = low frequency; RF = radiofrequency; SARmax = maximum specific absorption rate; W/kg = watts per kilogram; ECG = electrocardiogram; h = hour; EMF = electromagnetic field; Hz = hertz; mT = millitesla; µT = microtesla; PRF = pulse repetition frequency; LTE = Long-Term Evolution; 4G = fourth-generation mobile communication technology; V/m = volts per meter; RR = R-R interval; QTc = corrected QT interval; RRI = R-R interval series; SD = standard deviation; pVLF = very-low-frequency spectral power; pLF = low-frequency spectral power; pHF = high-frequency spectral power; ELF = extremely low-frequency; EEG = electroencephalography; MEG = magnetoencephalography; REM = rapid eye movement; PEMF = pulsed electromagnetic field; T/s = tesla per second; T = tesla; MRI = magnetic resonance imaging; CK-MB = creatine kinase-myocardial band; Ap = planetary amplitude index; RMSSD/rMSSD = root mean square of successive differences; pNN50 = percentage of successive normal-to-normal intervals differing by more than 50 ms; SBP = systolic blood pressure; DBP = diastolic blood pressure; BP = blood pressure; Kp = planetary geomagnetic index; CVC = cardiac vagal control; GMF = geomagnetic field; K = local geomagnetic K-index; SDNNi = mean of the standard deviations of normal-to-normal intervals for all 5-min segments; Dst = disturbance storm time index; ULF = ultra-low-frequency; mHz = millihertz.

Author, date	Exposure	Sample Size	Parameters evaluated	Outcome
Al-Hazimi et al. (2011) [24]	15-min GSM mobile phone call (1900 MHz; Nokia E90), with 15-min pre-call and post-call seated recordings	25 male students	HRV (time- and frequency-domain) and mean HR	During the call, HRV shifted toward greater parasympathetic and lower sympathetic modulation; mean HR did not change significantly. Specific changes included higher SDNN, VLF power, and HF power, with a lower LF/HF ratio during the call.
Andrzejak et al. (2008) [25]	20-min GSM mobile phone call (1800 MHz; Sony Ericsson K700i), with 20-min pre-call and post-call seated recordings	32 students	HRV (time- and frequency-domain)	During exposure and speaking, parasympathetic tone increased, and sympathetic tone decreased. In women, LF power increased, and LF/HF decreased during the call compared with pre/post call seated recordings.
Ahamed et al. (2008) [26]	30-min recordings under three conditions: no phone, phone in left shirt pocket near the chest, and phone held near the left ear; no incoming calls or messages were allowed during recording	14 males	Nonlinear HRV metrics (scaling exponent and sample entropy) and mean HR	HRV indices changed numerically with phone position: scaling exponent and sample entropy were higher when the phone was near the chest and lower when it was near the head, but none of these differences were statistically significant. Mean HR also did not change significantly.
Atlasz et al. (2006) [27]	Randomized RF vs. sham GSM exposure using a 900-MHz pulse-modulated mobile phone (2 W, SARmax 1.3 W/kg) for 10 min, with supine and standing measurements and repeated recovery assessments	35 adults	HR and HRV derived from ECG and infrared surface plethysmography	No significant differences in HR or HRV were found between RF-exposed and sham groups overall. A small subgroup (3/35) showed post-exposure HRV changes, especially in standing posture, but the overall study did not demonstrate a significant RF effect.
Ekici et al. (2016) [28]	Habitual mobile phone use grouped by daily call duration from billing records: none, <30 min/day, 30–60 min/day, and >60 min/day	148 adults	24-h Holter HRV indices and mean HR	HRV measures varied across phone-use duration groups, with greater daily call duration aligning with more sympathetic and less vagal HRV responses.
Parizek et al. (2023) [29]	5-min chest exposure at 1 cm to simulated 2400-MHz Wi-Fi and 2600-MHz LTE/4G signals (58 V/m), with control measurements before each exposure	30 adults	RR interval and HRV/autonomic indices (including HF-HRV and a sympathetic marker).	Small, frequency-dependent changes in autonomic markers during exposure showing reduced HF-HRV with 4G exposure and increased sympathetic marker with Wi-Fi exposure, without evidence of a large effect on cardiac timing.
Baldi et al. (2007) [30]	Extremely low-frequency pulsed EMF exposure using a Magnafield unit; 50-Hz field pulsed at 3 Hz, with two 20-min exposure periods separated by no-field periods; field intensity was approximately 1.57 mT at the pad and 4.21 µT at heart level	5 males	Holter ECG: HR/HRV indices	No formal statistical analysis was performed because of the small sample. Qualitatively, LF/HF ratio changes occurred mainly at transitions between exposure and nonexposure periods. Among the five subjects exposed to the same 3-Hz condition, the response pattern was similar but differed in magnitude. In the subject tested across multiple PRFs, the response appeared frequency-dependent, with the most noticeable variation at 3 Hz and a smaller similar pattern at 4 Hz, while 0.5 Hz showed little effect.
Borjanovic et al. (2005) [31]	Occupational 50-Hz magnetic field exposure in transformer substation workers, grouped by 12-h time-weighted average exposure: low 0.067 µT, medium 1.18 µT, and high 5.2 µT	59 males	ECG variables: HR, P-wave duration, PR interval, QRS duration, QT interval, and QTc	Only QTc differed significantly across exposure groups; adjusted mean QTc was similar in the low- and high-exposure groups but lower in the medium-exposure group, suggesting a non-linear exposure-response pattern. No significant differences were found in HR or the other ECG parameters.
Kurokawa et al. (2003) [32]	ELF alternating magnetic fields across three experiments using 50–1000 Hz fields at 20–100 µT, with continuous or intermittent exposure, circular or linear polarity, and exposure durations ranging from 2 min to 12 hours	50 adults	RR interval-derived HR and HRV indices: average RRI, SD of RRIs, pVLF, pLF, pHF, and pLF/pHF	Across all three experiments, no significant effects of exposure on HR or HRV were detected. The authors concluded that ELF magnetic fields of the type encountered in daily life did not acutely influence autonomic cardiovascular regulation under the tested conditions.
Sastre et al. (2000) [33]	Overnight intermittent 16 Hz magnetic field exposure in the beta1 EEG/MEG range at 28.3 µT under blinded laboratory conditions	18 men	Mean HR, HRV spectral measures, and timing of R waves around field on-off transitions	Exposure was associated with reduced low-frequency HRV power and a lower mean HR. Analyses of beat timing around field transitions found no evidence of a direct effect on the cardiac pacemaker, supporting a central nervous system mechanism rather than a direct cardiac one.
Graham et al. (2000) [34]	All-night intermittent 60 Hz circularly polarized magnetic field exposure at 28.3 µT versus sham in a randomized, double-blind, crossover design	46 adults	Overnight HR, HRV spectral measures, and polysomnographic sleep parameters	Mean HR was not significantly altered by exposure in either sex. In men, exposure was associated with reduced LF power over the night, suggesting altered autonomic balance. In women, HRV measures were not significantly altered, but exposure was associated with reduced REM sleep and trends toward lower sleep efficiency and total sleep time.
Graham et al. (2000) [35]	Pooled multistudy analysis of seven double-blind human overnight exposure studies of 60 Hz power-frequency magnetic fields, including intermittent and continuous exposure conditions at 28.3 µT and 127.3 µT	172 males	HRV spectral measures across pooled experimental datasets	The pooled analysis did not support a consistent, reproducible effect of magnetic field exposure on HRV across studies. HRV alterations were seen during intermittent 28.3 µT exposure only in studies that also involved hourly blood sampling via an indwelling catheter, suggesting that any field-related HRV effect may have depended on concomitant physiologic arousal or sleep disturbance rather than exposure alone.
Grote et al. (2007) [36]	Post-exercise pulsed EMF exposure after standardized physical stress tests using placebo, 0.005, 0.03, and 0.09 T/s conditions for 20 min	32 males	HRV, especially VLF power, and self-reported well-being during short-term recovery after exercise	Pulsed EMF exposure significantly affected post-exercise recovery of VLF power. Compared with placebo, 0.005 T/s was associated with faster recovery after physical strain. Responses at higher field strengths depended on baseline autonomic tone, with subjects who had lower baseline VLF power recovering more quickly than those with higher baseline VLF power. No effect on general well-being was observed.
Chakeres et al. (2003) [37]	Sequential static magnetic field exposure up to 8 T during simulated MRI conditions, with measurements obtained at 8, 6, 4.5, 3, and 1.5 T and matched out-of-field intervals	25 adults	HR, respiratory rate, systolic and diastolic blood pressure, oxygen saturation, core temperature, and ECG rhythm strips	No clinically significant changes in vital signs were observed with increasing field strength. The only statistically significant exposure-related change was a small rise in systolic blood pressure, averaging 3.6 mm Hg at 8 T. HR and post-exposure ECG rhythm strips did not show significant changes, although transient ECG artifacts increased with field strength.
Sert et al. (2010) [38]	30-min pre-exposure, during-exposure, and post-exposure assessments surrounding 30 min of exposure to a 1.5 T static magnetic field with the MRI system turned off to exclude RF and gradient field effects	30 males	HR/HRV; serum ions; troponin-I; CK-MB	During exposure, minimum, maximum, and average HR decreased, while several HRV indices increased, including rMSSD, pNN50, total power, power in the VLF, LF, and HF bands, suggesting increased autonomic modulation during static field exposure. Blood pressure did not change significantly, but respiration increased. After exposure, serum electrolytes, troponin-I, and CK-MB levels were higher than pre-exposure values.
Oinuma et al. (2002) [39]	Geomagnetic activity by daily ap-index using 24-h segments from 7-day Holter monitoring.	5 males	HR and HRV time-, frequency-, and geometric-domain measures	HRV decreased in a graded manner with higher geomagnetic activity, with significant reductions in overall variability measures and total power; mean HR did not differ significantly across ap-index categories.
Dimitrova et al. (2013) [40]	Naturally occurring solar and geomagnetic activity assessed using Ap-index values, with analysis of HRV before, during, and after geomagnetic disturbances with recordings over 1 year	14 adults	HR, RR intervals, SDNN, rMSSD, pNN50, LF power, HF power, LF/HF, SBP, DBP, and subjective psychophysiological complaints	HRV parameters varied from the day before through 3 days after weak geomagnetic storms. HR showed a trend of decreasing on the day of weak storms, while BP and subjective complaints increased after storm onset. Individual long-term recordings showed variable responses by storm intensity and timing, suggesting individualized adaptation rather than a uniform response.
Ozheredov et al. (2017) [41]	Geomagnetic activity indexed by Kp in relation to concurrent weather conditions over repeated 7-day ambulatory monitoring periods	197 adults	HR, SBP, DBP	Associations between geomagnetic activity and HR, SBP, and DBP were observed only under specific combinations of atmospheric pressure, temperature, and humidity, indicating weather-dependent magnetosensitivity rather than a uniform cardiovascular effect.
Ramishvili et al. (2023) [42]	Comparisons across quiet days and the initial, main, and restoration phases of geomagnetic storms using planetary K-index classification	61 males	HRV indices stratified by baseline autonomic state (high vs. low baseline HRV/CVC)	Responses to geomagnetic storms differed by baseline autonomic profile. Participants with high baseline HRV showed decreased HR and increased HRV in the main phase, consistent with a more adaptive parasympathetic response, whereas those with low baseline HRV showed patterns consistent with delayed sympathetic predominance and poorer adaptation.
Janashia et al. (2022) [43]	Experimental compensation of time-varying GMF components under different levels of outdoor geomagnetic activity, including quiet days, moderately disturbed days (K = 4), and geomagnetic storm days	25 males	HRV indices, including SDNN and LF power, assessed during baseline and GMF-compensation conditions	On quiet magnetic days, geomagnetic compensation did not meaningfully alter HRV. On K = 4 days, HR and stress index decreased, while SDNN and RMSSD increased; however, these changes were not statistically significant. During geomagnetic storm conditions, compensation was associated with significant increases in SDNN and LF power compared with baseline, suggesting that storm-related autonomic changes were modified when time-varying geomagnetic field components were compensated.
Caswell et al. (2016) [44]	Laboratory simulation of a sudden increase in geomagnetic activity compared with baseline recordings	20 adults	ECG-derived HRV frequency-domain measures, including LF power, HF power, and LF/HF ratio	Simulated geomagnetic activity was associated with significant increases in selected HRV frequency-domain parameters, particularly LF power and LF/HF ratio, compared with baseline recordings.
Gurfinkel et al. (2018) [45]	Laboratory simulation of a prerecorded geomagnetic storm: quiet magnetic field playback versus a 6-h storm replayed four times over 24 h	9 males	HRV (time/freq)	Relative to the quiet-field condition, the laboratory replay of a geomagnetic storm produced measurable HRV differences across the exposure period, consistent with autonomic modulation under storm-like magnetic variability.
Alabdulgader et al. (2018) [46]	Longitudinal ambulatory HRV monitoring in relation to changing solar and geomagnetic environmental measures over 5 months	16 females	HRV measures including inter-beat interval, total power, LF power, HF power, and LF/HF	Group-level autonomic activity varied with changes in the solar and geomagnetic environment. Increased solar wind intensity was associated with higher HR, while increases in cosmic rays, solar radio flux, and Schumann resonance power were associated with higher HRV and greater parasympathetic activity.
Mattoni et al. (2020) [47]	Continuous HRV monitoring over a 30-day period with concurrent geomagnetic and solar activity data	20 adults	HR, time-domain HRV, and frequency-domain HRV	Although several uncorrected associations were initially observed, most disappeared after correction for autocorrelation. The only surviving effects were a small increase in VLF-related HRV during higher local geomagnetic activity and a weak anticipatory decrease in HR before higher global geomagnetic activity. Overall, any effect appeared very small.
McCraty et al. (2017) [48]	Naturally occurring solar, cosmic ray, Schumann resonance, and local magnetic field variation assessed during continuous HRV monitoring over 31 days	10 adults	HRV variables correlated with solar wind speed, Kp, Ap, solar radio flux, cosmic ray counts, Schumann resonance power, and total magnetic field variation	Group-level HRV measures were significantly correlated with several environmental variables, including solar wind speed, Kp, Ap, solar radio flux, cosmic ray counts, Schumann resonance power, and total magnetic field variation. The direction of these associations varied by environmental period: solar wind speed showed negative correlations with several HRV measures during the post-storm period, while Schumann resonance power was positively associated with HRV early in the study but became negative or disrupted during storm and post-storm conditions. After normalization and removal of circadian rhythms, group HRV measures, including SDNNi, total power, and power in the VLF, LF, and HF bands, demonstrated a shared slow-wave oscillation of approximately 67 hours, suggesting possible synchronization between autonomic rhythms and time-varying geomagnetic or Schumann resonance activity.
Timofejeva et al. (2017) [49]	Local Earth magnetic field activity assessed over a two-week period using a synchronization analysis method comparing cardiac inter-beat intervals with local magnetic field data	20 adults	Cardiac inter-beat intervals; slow-wave HRV dynamics; synchronization between participant HRV patterns and local magnetic field data	A synchronization analysis method identified clusters of participants with similar HRV synchronization patterns relative to local magnetic field activity. Findings supported the possibility that slow-wave HRV rhythms can synchronize with local magnetic field variation, with synchronization patterns influenced by group/interpersonal factors.
Timofejeva et al. (2021) [50]	Local time-varying magnetic field intensity measured by synchronized magnetometers at five geographic sites during continuous ambulatory HRV monitoring; included a 15-min Heart Lock-In intervention performed simultaneously by participants	104 adults	HRV coherence; pairwise heart rhythm synchronization; HRV synchronization with local magnetic field power	Most groups showed increased HRV coherence during the 15-minute heart-focused coherence intervention. Pairwise heart rhythm synchronization among participants also increased during the intervention. In the HRV–local magnetic field analysis, synchronization was highest on the day the intervention was performed, with all groups showing positive HRV correlation with local magnetic field activity. These findings suggest increased cardiac coherence and within-group synchronization during the intervention, with concurrent HRV–magnetic field coupling, but do not establish causality.
Zenchenko et al. (2024) [51]	Multiyear naturally occurring geomagnetic field variation assessed using minute-value GMF vector data from the INTERMAGNET network during 100- or 120-min ECG recordings from 2012–2023	2 females; 403 ECG recordings	Minute HR time-series values; cross-correlation and wavelet similarity between HR oscillations and horizontal GMF components	Significant synchronization between minute HR values and at least one horizontal GMF component was observed in 40–53% of recordings. Wavelet analysis showed similarity between HR and GMF spectra, with synchronization most evident in the 8–13 min period range.
Zenchenko et al. (2025) [52]	Naturally occurring strong magnetic storms; 100-min ECG recordings obtained during September 2023, May 2024, and October 2024 events, with analysis of HR synchronization with ULF geomagnetic field variations in the 1–5 mHz range	2 females; 61 ECG recordings	Minute HR values and synchronization between HR oscillations and horizontal GMF components; Dst index; ULF oscillation amplitude	Biogeosynchronization between HR oscillations and geomagnetic field variations was observed in 69% of recordings. The probability of synchronization correlated with the Dst index but not ULF oscillation amplitude, and synchronization was most apparent during the recovery phase of magnetic storms.

RF and Mobile Phone Exposure

Several studies evaluated RF exposure from mobile phones. During short-term mobile phone calls, Al-Hazimi et al. [24] and Andrzejak et al. [25] both reported HRV changes consistent with greater parasympathetic and lower sympathetic modulation during exposure, although both authors noted that the act of speaking could not be excluded as a contributor to the observed effects. In contrast, Ahamed et al. [26] found only small, position-dependent changes in nonlinear HRV indices that were not statistically significant, and Atlasz et al. [27] reported no significant differences in HR or HRV between RF-exposed and sham conditions. Ekici et al. [28] examined habitual rather than acute mobile phone use and found that longer daily phone use was associated with lower HRV and a higher ratio of low-frequency to high-frequency power (LF/HF), suggesting greater sympathetic predominance among long-term users. Parizek et al. [29] reported frequency-specific autonomic effects during chest exposure to 2400 MHz Wi-Fi signals or 2600 MHz 4G signals, each for five minutes. They reported that Wi-Fi exposure was associated with a higher sympathetic HRV marker, while 4G exposure was associated with lower high-frequency HRV (HF-HRV), with neither exposure affecting the electrocardiogram (ECG)-determined RR intervals.

ELF and Pulsed EMF

In a small pilot study of pulsed EMF exposure, Baldi et al. [30] observed qualitative variation in the LF/HF ratio, particularly at transitions between exposure and non-exposure periods, and the response appeared pulse-repetition-frequency dependent; however, no statistical analysis was performed because of the very small sample size of only five males. A study of the effects of different ELF exposure times at the power-line frequency of 50 Hz by Borjanovic et al. [31] found that, of the ECG parameters measured, only the corrected QT interval (QTc) varied with exposure time, in a nonlinear manner. HR itself did not vary with exposure time. Kurokawa et al. [32] found no significant effects on HR or HRV across a wide range of ELF field frequencies (50-1000 Hz), intensities (20 to 100 µT), and exposure durations (two minutes to 12 hours). Contrastingly, Sastre et al. [33] reported reduced mean HR and lower LF power during overnight intermittent 16 Hz exposure at an intensity of 28.3 µT. They argued that the findings were more consistent with a central autonomic mechanism than with a direct cardiac pacemaker effect. Sleep and exercise-related studies also yielded variable results. Graham et al. [34] found that 60 Hz magnetic field exposure during sleep altered HRV in a sex-specific manner, with greater effects observed in men. However, their multi-study analysis did not demonstrate a consistent, reproducible HRV response across controlled laboratory protocols [35]. Grote et al. [36] found that pulsed EMF administered for 20 minutes after exercise influenced recovery of very-low-frequency (VLF) power, with responses depending on baseline autonomic tone.

Static Magnetic Field Exposure

Static magnetic field exposures ranging from 1.5 to 8 Tesla failed to cause any significant changes in HR or other vital signs, except for a small increase in blood pressure at the highest field strength [37]. In contrast, Sert et al. [38] reported that during 1.5 T exposure, HR parameters decreased while several HRV indices increased, with these changes returning toward baseline after exposure.

Geomagnetic Field Exposure

Geomagnetic exposure studies more often reported associations with cardiovascular variability, although the strength and consistency of those associations varied. Oinuma et al. [39] found a graded reduction in HRV with increasing geomagnetic disturbance, particularly in total power, and ultra-low-frequency (ULF) and VLF components, while mean HR did not differ significantly across ap-index categories. Dimitrova et al. [40] similarly reported that selected HRV parameters varied before, during, and after geomagnetic disturbances in healthy volunteers, although the direction and timing of responses differed across group-level and individual recordings. Ozheredov et al. [41] reported only weak associations between geomagnetic activity and hemodynamic measures, and these were most apparent under specific combinations of weather conditions.

Ramishvili et al. [42] reported that responses to geomagnetic storms differed according to baseline autonomic state, with participants who had higher baseline HRV showing patterns interpreted as better adaptation during the main phase of storms. Janashia et al. [43] found that compensation of time-varying geomagnetic field components did not significantly alter HRV during quiet magnetic days but produced significant increases in standard deviation of normal-to-normal intervals (SDNN) and LF power during geomagnetic storm conditions. Caswell et al. [44] reported that laboratory-simulated increases in geomagnetic activity were associated with significant increases in selected HRV frequency-domain parameters, particularly LF power and LF/HF ratio. Under laboratory conditions, Gurfinkel et al. [45] also simulated storm-like magnetic variability and observed measurable differences in cardiovascular variability metrics between quiet and storm exposure conditions.

In longer observational monitoring, Alabdulgader et al. [46] found that several HRV measures varied with solar and geomagnetic environmental changes over time. By contrast, Mattoni et al. [47] found that most initially significant associations between HRV and geomagnetic or solar variables disappeared after correction for autocorrelation, leaving only small residual effects. McCraty et al. [48] also reported significant correlations between HRV variables and solar, cosmic ray, Schumann resonance, and local magnetic field measures during 31 days of continuous monitoring in healthy adults. After normalization and removal of circadian rhythms, participants’ HRV rhythms appeared synchronized across the monitoring period, suggesting possible coupling between autonomic activity and time-varying environmental magnetic field activity.

Several studies further examined synchronization between HR or HRV and local or geomagnetic field variation. Timofejeva et al. [49] developed and applied a synchronization analysis method in 20 healthy participants and reported that slow-wave HRV rhythms could synchronize with local magnetic field data. In a later global study, Timofejeva et al. [50] examined continuous ambulatory HRV recordings from participants across five geographic sites and included a simultaneous 15-minute heart-focused coherence intervention. During the intervention, most groups showed increased HRV coherence, and participants’ heart rhythms became more synchronized with those of other members of their local group. HRV synchronization with local magnetic field activity was also greatest on the day of the intervention. Zenchenko et al. [51] analyzed repeated ECG recordings from two healthy female volunteers and found synchronization between minute-level HR oscillations and horizontal geomagnetic field components in a substantial proportion of recordings, with the strongest synchronization occurring in the 8-13 min period range. In a subsequent study during strong magnetic storms, Zenchenko et al. [52] reported synchronization between HR oscillations and ULF geomagnetic field variations in most recordings, with synchronization most apparent during the recovery phase of magnetic storms.

Methodological factors and variability

The included studies were methodologically heterogeneous. Exposure frequencies ranged from LF and power-frequency protocols to 2600-MHz RF signals; reported magnetic-field magnitudes ranged from microtesla levels to static fields of 8 T; and exposure duration ranged from several minutes to overnight sessions or months of environmental monitoring. Some evaluated brief, controlled exposures during mobile phone use or laboratory sessions, whereas others examined habitual mobile phone use, overnight sleep exposure, post-exercise recovery, geomagnetic storm periods, local magnetic field variation, HRV synchronization, or repeated monitoring during changing geomagnetic conditions. As a result, the studies did not assess a single uniform exposure model, which likely contributed to the inconsistency of findings across the literature.

The outcomes measured also differed substantially. While many studies focused on conventional HRV time- and frequency-domain measures, others emphasized nonlinear HRV indices, ECG intervals, blood pressure, broader physiologic variables, HRV coherence, or minute-scale HR synchronization with geomagnetic field variation [26,31,37,38,41,48-52]. This diversity in endpoint selection further limits direct comparison across studies and complicates interpretation of the overall evidence base. In addition, several studies contained design-related factors that complicate interpretation, including the potential influence of speaking during mobile phone exposure, the known dependence of HRV on sleep physiology, differences in baseline autonomic tone, small samples despite repeated recordings, interpersonal or group-coherence conditions, and the challenge of properly accounting for autocorrelation in geomagnetic time-series analyses [24,25,34-37,42,47-52].

Gaps and limitations

Many included studies were limited by relatively small sample sizes and short-term exposure paradigms, which reduce precision and make subtle physiologic effects difficult to interpret with confidence [24-27,30,32,37,38,40,43-45,48,49,51,52]. Although several geomagnetic studies used repeated recordings or longer observational monitoring, some relied on very small numbers of participants, limiting generalizability despite the large number of repeated measurements [46,48,49,51,52]. This limitation was most pronounced in studies that included as few as two to five participants [39,51,52].

The literature was also marked by substantial variation in exposure protocols, field strengths, exposure durations, geomagnetic indices, local magnetic field measurements, synchronization methods, environmental covariates, behavioral conditions, and analytic methods [24-52]. Because of this variability, the studies do not readily support simple cross-study synthesis, even when they nominally address similar exposure categories.

Summary of evidence

Overall, the current literature does not demonstrate a single consistent or reproducible effect of field exposure on HR or HRV in healthy adults. Several studies reported no significant effect, while others identified only small or context-dependent changes [26,27,32,35,37,47].

Studies of man-made EMF exposure yielded mixed findings. Some reported no measurable effect on HR or HRV, whereas others described transient or exposure-specific alterations in autonomic markers rather than a stable or uniform physiologic pattern [24-38].

Geomagnetic studies more frequently reported associations with HRV or hemodynamic variation; however, these effects were generally modest, methodologically heterogeneous, and less convincing when evaluated with more rigorous analytic approaches [39-52]. Collectively, these studies suggest that HR or HRV may vary with geomagnetic or local magnetic field conditions, particularly in studies examining storm periods, long-term monitoring, field compensation, or HR/HRV synchronization [40,43,44,48-52]. However, these findings remain context-dependent and should be interpreted as evidence of possible subtle autonomic modulation rather than a consistent or clinically meaningful effect across healthy adults.

Discussion

The findings of this scoping review suggest that geomagnetic and man-made EMF exposures may influence HRV under some conditions, but the direction, magnitude, and reproducibility of these effects remain inconsistent. This interpretation is consistent with prior background literature, which has described possible cardiovascular effects of both geomagnetic and man-made EMF exposures, while also emphasizing the inconsistency of the available evidence [8,12,13]. In the present review, studies of man-made EMF exposure more often showed null findings or transient changes in selected autonomic markers, whereas geomagnetic studies more often reported associations with HRV or hemodynamic variation, although these effects were generally modest and not uniformly observed across studies [24-52]. Several geomagnetic and local magnetic field studies suggest that HR and HRV may be responsive to naturally occurring geomagnetic variation, local magnetic field changes, or storm-like exposure conditions [40,43,44,48-52]. However, statistical significance in an isolated HRV index should not be equated with clinical significance. As these studies were primarily designed to assess short-term physiological responses rather than long-term cardiovascular outcomes, it remains unclear whether the reported effect sizes exceed normal daily variability. Consequently, without evidence of persistent symptoms or arrhythmias, these transient HRV fluctuations cannot currently be interpreted as clinically harmful.

The findings related to man-made EMF exposure were also mixed, but the pattern differed from the geomagnetic literature. Several acute exposure studies found no significant effects on HR or HRV, while others described transient changes in selected autonomic markers during or shortly after exposure [24-27,30,32,37,38]. This is consistent with prior reviews, which have also concluded that the cardiovascular literature on EMF exposure is inconsistent and highly dependent on exposure conditions and study design [12,13]. In the present review, studies reporting measurable effects generally described changes that were transient, parameter-specific, or dependent on contextual factors such as speaking during a phone call, sleep-state physiology, field frequency, or baseline autonomic tone, rather than demonstrating a stable and reproducible autonomic effect across studies [24,25,34-36].

Differences across studies likely reflect real differences in the exposure model and study context rather than simple disagreement in results. Acute laboratory studies evaluated brief, isolated exposures during mobile phone use, sleep, post-exercise recovery, static-field testing, or simulated geomagnetic disturbance [24-27,29,30,32-38,44,45]. In contrast, many geomagnetic studies assessed naturally occurring environmental changes over days, months, or years, often under less controllable conditions [39-43,46-52]. Several geomagnetic studies did not primarily examine conventional exposure-response effects but instead evaluated synchronization between HR or HRV rhythms and local or geomagnetic field variation [48-52]. These are not equivalent experimental situations, and it is therefore unsurprising that the literature does not support a single uniform physiologic pattern. Instead, the present review suggests that any observed autonomic effects are likely to be exposure-specific and context-dependent.

The evidence for geomagnetic effects warrants particularly cautious interpretation. Several included studies reported reductions in HRV or changes in hemodynamic variables during geomagnetically disturbed periods or storm conditions [39-45,52]. This direction of effect is broadly compatible with literature linking geomagnetic activity to blood pressure variation and wider cardiovascular concerns [1,2,5,6]. However, the included geomagnetic studies varied considerably in design, analytic method, and control of environmental complexity. Across this body of evidence, some studies examined natural geomagnetic storms or storm phases, while others evaluated simulated geomagnetic disturbance, compensation of time-varying geomagnetic field components, local magnetic field fluctuations, long-term monitoring, or synchronization between HR/HRV rhythms and geomagnetic oscillations [40,42-52]. This breadth supports the possibility of geomagnetic-cardiac interaction, but it also increases heterogeneity and limits direct comparison across studies.

Within the geomagnetic literature, several studies evaluated not only changes in conventional HRV indices, but also whether cardiac rhythms showed temporal alignment with local or geomagnetic field variation. This synchronization-focused evidence complements the storm-period, field-compensation, and long-term monitoring studies by suggesting that geomagnetic or local magnetic field variation may be associated with slow-wave cardiac rhythm dynamics under certain conditions. McCraty et al. [48] and Timofejeva et al. [49,50] reported synchronization between HRV rhythms and local magnetic field activity, while Zenchenko et al. [51,52] described synchronization between minute-level HR oscillations and horizontal geomagnetic field components. However, temporal synchronization alone cannot establish a causal relationship between geomagnetic fluctuations and cardiac rhythms. These findings remain controversial and carry less evidentiary weight than controlled experiments, as they are highly susceptible to unmeasured confounding, such as shared circadian trends, and statistical artifacts. For example, most of the initially significant associations reported by Mattoni et al. [47] lost significance once adequately corrected for autocorrelation [47-52].

Methodological limitations remain central to interpretation. Many studies had small sample sizes, and although several geomagnetic studies included repeated recordings or longer monitoring periods, some relied on very small numbers of participants, which limits generalizability [40,51,52]. Repeated-measures designs can provide useful within-person information, but they do not eliminate the need for larger sample sizes when evaluating reproducibility across broader populations. In addition, geomagnetic studies are particularly vulnerable to analytic challenges such as autocorrelation, time-lag selection, environmental confounding, and multiple comparisons [41,46-52]. These concerns are important because some associations may appear stronger before appropriate adjustment for the time-series structure. This is especially relevant for studies correlating HRV with geomagnetic, solar, or local magnetic field variables over time, where apparent associations may be influenced by shared temporal patterns, circadian rhythms, environmental factors, or behavioral conditions [46-52].

The practical implications of these findings remain limited. The available evidence does not support a consistent or clinically meaningful cardiovascular risk associated with exposure to geomagnetic or man-made EMFs in healthy adults. However, the geomagnetic literature suggests that subtle changes in HR or HRV may occur under certain environmental or experimental conditions, particularly during geomagnetic disturbances or when local magnetic field variations are examined in relation to slow cardiac rhythm dynamics [39-52]. Future studies should use larger sample sizes, standardized HRV acquisition and processing methods, prespecified geomagnetic exposure metrics, appropriate correction for autocorrelation, and careful control for weather, circadian, behavioral, and baseline autonomic factors. Future work should also distinguish between conventional HRV measures, hemodynamic outcomes, HRV coherence, and HR/HRV synchronization outcomes, as these may represent related but distinct physiologic responses.

## Conclusions

This scoping review found no single, consistent, or reproducible effect of geomagnetic or man-made EMF exposure on HR or HRV in healthy adults, although several studies reported transient or context-dependent changes in selected autonomic markers. Geomagnetic studies more often reported associations with HRV or hemodynamic variation than studies of man-made EMF exposure, but these effects were generally modest, methodologically heterogeneous, and in some cases substantially attenuated after more rigorous analytic correction. Taken together, the available evidence suggests possible subtle autonomic or rhythm-related responses under specific exposure conditions, particularly in geomagnetic and local magnetic field studies, but does not establish a consistent, clinically meaningful effect across healthy adults.

This body of evidence is limited by small and often non-independent samples, substantial heterogeneity in exposure protocols and HRV methodology, and, for many geomagnetic studies, inadequate control of autocorrelation and environmental confounding - limitations reflected in the high proportion of studies rated as high methodological concern in this review. Future research should prioritize larger samples, standardized and prespecified exposure and HRV metrics, rigorous correction for time-series dependence, and preregistered replication of key findings, particularly those involving geomagnetic storms and HR/HRV synchronization.

## Appendices

**Table 4:** Joanna Briggs Institute critical appraisal checklist for randomized controlled studies. RCT: randomized controlled trial.

Study	Was true randomization used for assignment of participants to groups?	Was allocation to groups concealed?	Were groups similar at baseline?	Were participants blind to group assignment?	Were those delivering the exposure/intervention blind to group assignment?	Were groups treated identically other than the exposure/intervention of interest?	Were outcome assessors blind to group assignment?	Were outcomes measured in the same way for all groups?	Were outcomes measured in a reliable way?	Was follow-up complete and, if not, were reasons for loss to follow-up described and analyzed?	Were participants analyzed in the groups to which they were randomized?	Was appropriate statistical analysis used?	Was the trial design appropriate, and were deviations from a standard RCT accounted for?	Overall methodological concern
Atlasz et al. (2006) [27]	Unclear	Unclear	Unclear	Yes	Unclear	Yes	Unclear	Yes	Yes	Yes	Yes	Yes	Yes	Moderate
Kurokawa et al. (2003) [32]	Yes	Unclear	Yes	Yes	Unclear	Yes	Unclear	Yes	Yes	Yes	Yes	Yes	Yes	Moderate
Sastre et al. (2000) [33]	Unclear	Unclear	Unclear	Yes	Unclear	Yes	Unclear	Yes	Yes	Yes	Yes	Yes	Yes	Moderate
Graham et al. (2000) [34]	Yes	Unclear	Yes	Yes	Yes	Yes	Yes	Yes	Yes	Yes	Yes	Yes	Yes	Low
Gurfinkel et al. (2018) [45]	Unclear	Unclear	Yes	Yes	Unclear	Yes	Unclear	Yes	Yes	Yes	Yes	Unclear	Unclear	Moderate

**Table 5:** Joanna Briggs Institute critical appraisal checklist for quasi-experimental studies.

Study	Was it clear what was the cause and what was the effect?	Was there a control or comparison condition?	Were participants included in comparisons similar?	Were participants treated similarly other than the exposure/intervention of interest?	Were there multiple measurements before and after the exposure/intervention?	Were outcomes measured in the same way for all comparisons?	Were outcomes measured in a reliable way?	Was follow-up complete and, if not, were differences in follow-up described and analyzed?	Was appropriate statistical analysis used?	Overall methodological concern
Al-Hazimi et al. (2011) [24]	Yes	No	Yes	No	No	Yes	Unclear	Yes	No	High
Andrzejak et al. (2008) [25]	Yes	No	Yes	No	No	Yes	Yes	Yes	Yes	High
Ahamed et al. (2008) [26]	Yes	No	Yes	No	No	Yes	Yes	Yes	No	High
Baldi et al. (2007) [30]	Yes	Unclear	Yes	No	No	Yes	Yes	Yes	No	High
Parizek et al. (2023) [29]	Yes	Yes	Yes	No	No	Yes	Yes	Yes	Unclear	Moderate
Grote et al. (2007) [36]	Yes	Yes	Yes	Yes	Yes	Yes	Yes	Unclear	Yes	Low
Chakeres et al. (2003) [37]	Yes	Yes	Yes	Yes	Yes	Yes	Unclear	Yes	Yes	Low
Sert et al. (2010) [38]	Yes	No	Yes	No	No	Yes	Unclear	Yes	Unclear	High
Janashia et al. (2022) [43]	Yes	Yes	Yes	No	No	Yes	Yes	Yes	No	High
Caswell et al. (2016) [44]	Yes	Yes	Yes	Yes	No	Yes	Yes	Yes	Unclear	Moderate

**Table 6:** Joanna Briggs Institute critical appraisal checklist for analytical cross-sectional studies.

Study	Were the criteria for inclusion in the sample clearly defined?	Were objective, standard criteria used to define participants as healthy or asymptomatic?	Was exposure measured in a valid and reliable way?	Were outcomes measured in a valid and reliable way?	Were confounding factors identified?	Were strategies to address confounding factors stated?	Was appropriate statistical analysis used?	Were the study subjects and setting described in sufficient detail?	Overall methodological concern
Ekici et al. (2016) [28]	Yes	Yes	Unclear	Yes	Yes	Unclear	Yes	Yes	Moderate
Borjanovic et al. (2005) [31]	Yes	Unclear	No	Yes	No	No	No	Yes	High
Graham et al. (2000) [35]	Yes	Yes	Yes	Yes	Yes	Yes	Unclear	Yes	Moderate
Oinuma et al. (2002) [39]	Yes	Unclear	Yes	Yes	No	No	No	Yes	High
Dimitrova et al. (2013) [40]	Unclear	Unclear	Yes	Yes	Unclear	No	No	Yes	High
Ozheredov et al. (2017) [41]	Yes	Yes	Yes	Yes	Yes	Unclear	No	Yes	High
Ramishvili et al. (2023) [42]	Yes	Yes	Yes	Yes	Unclear	Unclear	Yes	Yes	Moderate
Alabdulgader et al. (2018) [46]	Yes	Yes	Yes	Yes	Unclear	Unclear	Yes	Yes	Moderate
Mattoni et al. (2020) [47]	Yes	Yes	Yes	Yes	Yes	Yes	Unclear	Yes	Low
McCraty et al. (2017) [48]	Yes	Yes	Yes	Yes	No	No	No	Yes	High
Timofejeva et al. (2017) [49]	Unclear	Unclear	Yes	Unclear	No	No	No	Unclear	High
Timofejeva et al. (2021) [50]	Yes	Yes	Yes	Yes	Yes	No	Unclear	Yes	Moderate
Zenchenko et al. (2024) [51]	No	Unclear	Yes	Yes	Unclear	No	Unclear	Yes	High
Zenchenko et al. (2025) [52]	No	Unclear	Yes	Yes	Unclear	No	No	Yes	High
